# What Is Your Diagnosis? Esophageal and Gastric Washes in a Pet Moellendorff's Rat Snake

**DOI:** 10.1111/vcp.70073

**Published:** 2025-11-03

**Authors:** Lina C. Bilhalva, Priscila B. S. Serpa, Natalia J. Strandberg, Sriveny Dangoudoubiyam, Craig A. Thompson, Andrea P. Santos

**Affiliations:** ^1^ Department of Comparative Pathobiology Cummings School of Veterinary Medicine at Tufts University North Grafton Massachusetts USA; ^2^ Department of Comparative Pathobiology Purdue University College of Veterinary Medicine West Lafayette Indiana USA; ^3^ Department of Population Medicine and Diagnostic Sciences Cornell University College of Veterinary Medicine Ithaca New York USA; ^4^ Department of Biomedical Sciences and Pathobiology Virginia‐Maryland College of Veterinary Medicine Blacksburg Virginia USA

## 1 Case Presentation

A 6‐year‐old female Moellendorff's rat snake ( *Orthriophis moellendorffi* ), weighing 0.8 kg, was presented to the Purdue University Veterinary Hospital for evaluation of open‐mouth breathing, hypersalivation, and gurgling respiratory sounds. The owner reported that the snake had been quarantined in the serpentarium 1 week prior due to excessive oral mucus, though its appetite remained normal. The owner also disclosed maintaining approximately 50 snakes but provided no further husbandry details. On physical examination, the snake was alert and responsive but exhibited dyspnea. A laryngeal swab was collected for bacterial culture and sensitivity testing; after 5 days, aerobic culture yielded no growth. Ten days later, with no clinical improvement, the snake returned for reevaluation. Bloodwork was unremarkable, but radiographs revealed esophageal dilation with gas and increased soft tissue opacity caudal to the heart, suggestive of esophageal wall thickening or a mass. No radiographic evidence of respiratory disease was observed. Gastric and esophageal washes were obtained for cytologic analysis (Figure 1A,B).

**FIGURE 1:**
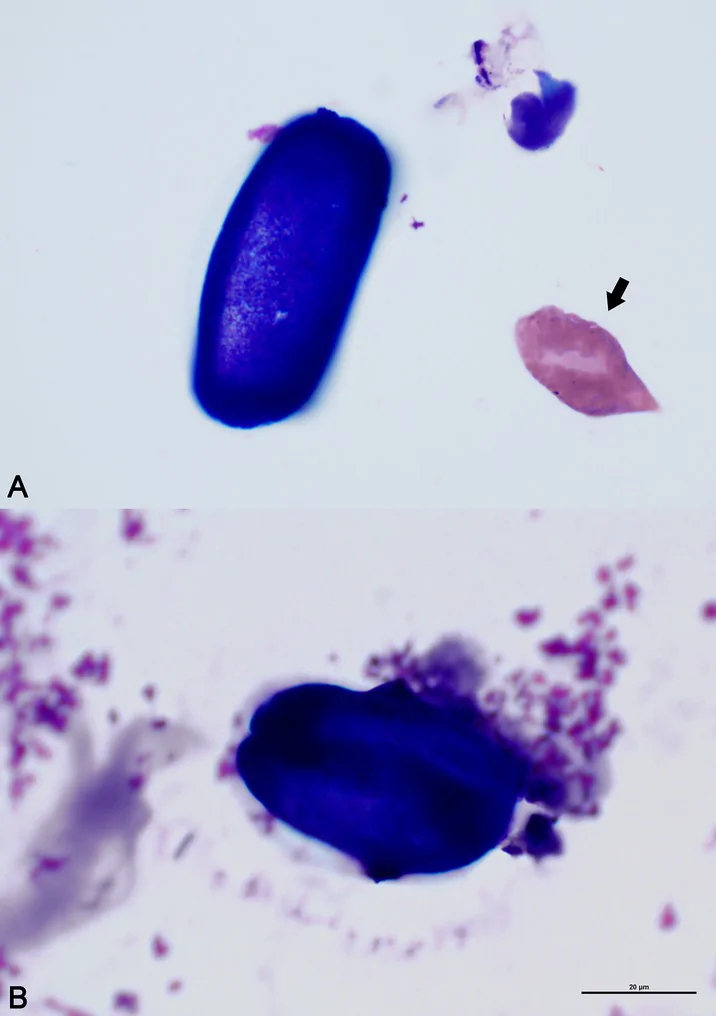
Cytologic preparation showing the basophilic oval structures in (A) esophageal and (B) gastric washes of a pet Moellendorff's rat snake. The black arrow points to a heterophil. Modified Wright stain, ×100 objective, bar = 20 μm.

## 2 Cytologic Description

Both samples exhibited similar features. The samples were of low cellularity, consisting mainly of poorly preserved inflammatory cells and scant erythrocytes in the background. The leukocyte differential consisted of approximately 70% heterophils, 25% macrophages, and 5% lymphocytes. Rare basophils and plasma cells were identified on scanning, along with numerous unremarkable ciliated columnar cells and goblet cells. Notably, sparse thin‐walled oval structures (25–35 μm wide × 45–50 μm long) were present. Frequently, a discernible, deeply basophilic granular material consistent with larval forms was seen inside these structures. The cytologic interpretation was mild heterophilic inflammation with larvated ova morphologically compatible with nematode eggs (likely *Strongyloides* /*Rhabdias* spp.).

## 3 Additional Results

Smears of the esophageal and gastric washes underwent molecular testing at the University of Florida's Zoological Medicine and Wildlife Disease Laboratory. Genomic DNA was extracted and amplified via PCR using primers NC5 (5′‐GTA GGT GAA CCT GCG GAA GGA TCA TT‐3′) and NC2 (5′‐TTA GTT TCT TTT CCT CCG CT‐3′), which target conserved regions flanking the ITS1‐5.8S‐ITS2 ribosomal complex in nematodes [1]. The amplified PCR products were submitted for Sanger sequencing. Following quality trimming and assembly, a final consensus sequence of 581 bp was submitted to GenBank under accession number PQ303691. A comparison with the database revealed similarity to a previously described *Strongyloides* sp. (97.7%; GenBank entry: OQ107246.1) in a colony of captive colubrids [2].

Despite treatment with fenbendazole (for endoparasites) and metronidazole (for potential secondary bacterial infection), the snake's condition deteriorated, necessitating euthanasia. No postmortem examination or fecal analysis was conducted.

## 4 Discussion

*Strongyloides* are nematodes belonging to the family Strongyloididae [3]. At least 50 species have been described within this genus [4], infecting mammals, birds, reptiles, and amphibians [5]. Transmission occurs when larvae penetrate the host's skin or oral mucosa, enter systemic circulation, and migrate to the lungs [3]. They access the oral cavity through the trachea, where they can be ingested and subsequently enter the gastrointestinal tract [3]. However, both the Strongyloididae and Rhabdiasidae families have the potential to cause clinical signs associated with respiratory disease due to larval migration or adult parasites residing in the respiratory tissue, respectively [3]. Infected snakes often exhibit open‐mouth breathing, mucous accumulation around the nares and glottis, and may develop severe pneumonia with secondary bacterial infection [3]. Because both families produce morphologically similar larvated eggs in feces [3], molecular analysis was critical for an accurate etiologic diagnosis in this case. The amplified sequence showed the highest similarity to a potential novel *Strongyloides* species recently identified in captive colubrids from central Florida, USA [2]. In that study, affected snakes frequently demonstrated esophageal thickening, with nematodes sometimes visible in the esophagus and stomach at various life stages [2]. The esophageal soft tissue opacity observed radiographically likely represented a helminth infection focus, though this couldn't be confirmed histologically. The snake's clinical signs may have reflected tracheal compression by the esophageal mass or dysphagia misinterpreted as respiratory distress.

Although *Strongyloides* infections are common in snakes, this represents the first reported case in *O. moellendorffi* . While not contradictory, the diagnosis was unexpectedly achieved through cytologic examination of esophageal and gastric washes. The sequence's strong similarity to a potentially novel and highly pathogenic *Strongyloides* species [2] raises concerns about transmission risks in captive environments. This finding underscores the value of molecular methods for identifying emerging parasitic threats in reptile collections.

## Conflicts of Interest

The authors declare no conflicts of interest.

## References

[1] R. B. Gasser , L. E. Stewart , and R. Speare , “Genetic Markers in Ribosomal DNA for Hookworm Identification,” Acta Tropica 62 (1996): 15–21. 10.1016/s0001-706x(96)00015-0 8971275

[2] E. A. Graham , E. W. Los Kamp , N. M. Thompson , et al., “Proliferative Strongyloidiasis in a Colony of Colubrid Snakes,” Veterinary Pathology 61 (2024): 109–118. 10.1177/03009858231186391 37458163

[3] H. D. S. Walden , E. C. Greiner , and E. R. Jacobson , “Parasites and Parasitic Diseases of Reptiles,” in Infectious Diseases and Pathology of Reptiles: Color Atlas and Text, 2nd ed., ed. E. R. Jacobson and M. M. Garner (CRC Press, 2020), 859–968.

[4] V. L. T. Mati and A. L. de Melo , “Some Aspects of the Life History and Morphology of *Strongyloides ophidiae* Pereira, 1929 (Rhabditida: Strongyloididae) in *Liophis miliaris* (Squamata: Dipsadidae),” Neotropical Helminthology 8 (2014): 203–216.

[5] M. Viney , “Strongyloides,” Parasitol 144 (2017): 259–262. 10.1017/S0031182016001773 PMC5364833 27759560

